# The Majority of the Migrant Factory Workers of the Light Industry in Shenzhen, China May Be Physically Inactive

**DOI:** 10.1371/journal.pone.0131734

**Published:** 2015-08-05

**Authors:** Jing Liu, Yu Cheng, Joseph T. F. Lau, Anise M. S. Wu, Vincent W. S. Tse, Shenglai Zhou

**Affiliations:** 1 Research Centre for Healthcare Management, School of Economics and Management, Tsinghua University, Beijing, China; 2 School of Sociology and Anthropology, Sun Yat-sen University, Guangzhou, China; 3 Center for Medical Anthropology and Behavioral Health, Sun Yat-sen University, Guangzhou, China; 4 Division of Health Improvement, JC School of Public Health and Primary Care, The Chinese University of Hong Kong, Prince of Wales Hospital, Shatin, NT, Hong Kong, China; 5 The Chinese University of Hong Kong Shenzhen Research Institute, Shenzhen, China; 6 Department of Psychology, Faculty of Social Sciences, University of Macau, Macao, China; 7 Department of Educational Psychology, The Chinese University of Hong Kong, Hong Kong, China; 8 Beijing Anzhen Hospital, Beijing, China; Old Dominion University, UNITED STATES

## Abstract

Physical inactivity is a strong risk factor of non-communicable diseases (NCD). In China, there are 250 million migrant factory workers, who are susceptible to physical inactivity and hence NCD because of work nature and setting. With random stratified sampling, 807 such workers of the light industry were recruited in Shenzhen, China and completed a self-administered questionnaire with informed consent. The prevalence of inadequate physical activity (defined according to the World Health Organization’s recommendation on level of moderate/vigorous physical activity) was 95.4%. Of all participants, 69.1% showed “a very low level of physical activity” (VLLPA), defined as ≤30 minutes of weekly moderate/vigorous physical activity, which was significantly associated with female sex (Odds ratio [OR]=1.65), lower education level (OR=0.10 to 0.33, primary education as the reference group) and married status (OR=0.63, single status as the reference group). Adjusted for these factors, perceived social support (Adjusted OR=0.87) was negatively associated with VLLPA, while job stress due to workload, which was significant in the univariate analysis (OR=0.98), became non-significant (*p*=0.184). Significant interaction between perceived social support and perceived job stress onto VLLPA was found (*p*=0.044), implying that the negative association between job stress and VLLPA, which might reflect a potential response to cope with stress by performing exercises, was stronger among those with weaker social support. The extremely low level of physical activity rings an alarm, as it implies high risk of NCD, and as there are no existing programs promoting physical activity in this group. Interventions need to take into account social support, potential coping to job stress, and structural factors of the factory setting, while involving factories’ management.

## Introduction

Prevalence of non-communicable diseases (NCD) keeps increasing sharply in China [1–2]. For instance, it was estimated that there were 92.4 million adult diabetic patients living in the country [3]. According to the data released by the China CDC, the prevalence of hypertension among adults aged 18 years and older reached 33.5% in 2010 in China [4]. It is warranted to promote healthy lifestyle, as there are strong evidences that it is protective of NCD [5–6].

Physical activity is one of the most important health-related behaviors that are strongly associated with population health [7–8]. There are clear evidences that physical activity can effectively prevent cardiovascular disease, diabetes, cancer, hypertension, bone and joint diseases, depression and other chronic diseases [5, 9–12]. Importantly, there are reports that health promotion programs can effectively increase physical activity in various populations [13–14]. Despite the importance, the average time spent on physical activity per week among adults in China decreased by 32% from 1991 to 2006 [15]. It was suggested that phenomenal internal migration from rural to urban areas contributed to increasingly sedentary lifestyle in China [16].

Factory workers make up a large proportion of China’s internal migrants. Health workers hence need to pay special attention to their health-related behaviors, especially among those who migrated from rural areas to large cities in China [17]. It is estimated that there is a total of more than 250 million such workers [18], most of them are living and working in large coastal Chinese cities. As an illustration, a study showed that about one-fourth (26.6 out of 104 millions) of the population in Guangdong province was made up of internal migrant workers [19]. Migrant factory workers tend to be relatively young and come from rural areas [20]. With the large population size, their health problems may contribute substantially to the country’s disease burden. Our literature review however, did not locate any intervention promoting physical activity in this special and huge population in China.

It is known that settings determine health-related behaviors [21]. A high percentage of the internal migrants of costal Chinese cities such as Guangdong are made up of factory workers [19]. Factories in China, especially those of the light industry, may not be a setting that is supportive of healthy life-style, as the majority of their workers have to spend long hours working in a very confined environment and most of them live in the factories’ dormitories. Besides, factory workers tend to have lower education levels and low health literacy [22–24]. They are hence potentially exposed to unhealthy behaviors and hence high risk of NCD. Their health needs may have been overlooked as there is a dearth of studies investigating health-related behaviors of migrant factory workers in China. This study hence focused on migrant factory workers of the light industry.

To promote physical activity, we need to consider mental health status as previous studies have shown that it can be positively or negatively associated with health-related behaviors. Some researchers have reported that perceived stress [25–29], and mental health problems (e.g. depression and anxiety [28,30–38]) were negatively associated with physical activity. In contrast, other researchers have reported positive associations between various types of job stress and level of physical exercise [25–27], possibly because physical exercise is used as an adaptive response to cope with job stress and to relieve stress [28,39,40]. Migrant workers tend to lead a very stressful life as they are away from home and need to adapt to their new urban lifestyle [41,42]. It is warranted to investigate whether job stress would be associated with higher or lower levels of physical activity among these migrant factory workers. Such information facilitates planning programs for stress reduction and promotion of physical activity.

Significant and positive associations between perceived social support and frequency of physical exercise have been reported [29,43–46]. Perceived social support is also a potential moderator of the association between job stress and physical activity. Significant moderation effect implies that the strength of association between job stress level and physical activity level varies according to the strength of social support. As a coping resource [47], social support can potentially buffer negative effects of stress [48] and amplify positive coping responses [49]. In literature, research on the moderation effect of social support onto associations between perceived stress and health-related behaviors (e.g. alcohol consumption, smoking and physical activity) has shown mixed results [50–52].

Lack of time is a commonly cited reason for being physically inactive [53,54]. In order to increase income, migrant factory workers in China tend to have long average working hours, often 10 or more hours per day, as over-time working hours are paid higher than regular working hours [55–59]. Such long working hours may potentially lead to sedentary life-style and lack of time to perform physical exercise, and may contribute to physical inactivity among factory workers. There is however, no study looking at association between working hours and physical activity among migrant factory workers.

Considering potential importance of promoting physical activity among migrant factory workers in China, the present study investigated the prevalence of physical inactivity among factory workers who were internal migrants living in Shenzhen, Guangdong province, southern China. In this study, inadequate physical activity was defined, according to the Global Recommendation on Physical Activity for adults from the World Health Organization (WHO), as less than 150 minutes of moderate or vigorous physical activities per week (WHO, 2010). In addition, associations between various types of factors (socio-demographic factors, type of workers, work hours, perceived job stress due to workload and perceived social support) and a very low level of moderate/vigorous physical activity (VLLPA), which was defined as ≤30 minutes of moderate/vigorous physical activity per week), were investigated.

We hypothesized that i) long working hour, ii) high level of perceived job stress due to workload, and iii) lower level of perceived social support would be positively associated with VLLPA. We hence contended that job stress due to workload is a risk factor of a VLLPA. In addition, we tested the hypothesis that perceived social support would moderate the association between perceived job stress and VLLPA (i.e. presence of a significant statistical interaction). To our knowledge, no study has reported physical activity level and associated factors among internal migrant factory workers in China.

## Methods

### Sampling

The study was conducted in Shenzhen in southern China. Of the 10.4 million residents in Shenzhen, 77% were internal migrants and 46% were factory workers [60]. In 2011, there were 5692 factories registered in Shenzhen, of which 41.7% belonged to the light industry (e.g. electronics and garment) [60]. The city ranked fourth in GDP among all cities in China in 2012 [61]. Three factories in Shenzhen were contacted by the researchers through referrals of personal contacts, and were invited to participate in this study; random sampling method was hence not used. The factors produced high technology products (4,964 workers), television sets (1,560 workers) and electronic products (1,761 workers). The study population hence only included internal migrant factory workers of the light industry. It did not include migrant workers of the heavy industry, nor those of the construction industry; such workers have higher levels of work-related physical activity. Internal migrants were defined as those coming from other parts of China and did not hold an official registered resident status (Hu-Kou) in Shenzhen.

Stratified randomized sampling methods were used. Eligible participants were classified into six strata: three work-type subgroups (production line workers, office workers, and others) times two age groups (≤30 and >30). The name lists within each stratum were sorted alphabetically and respectively 15% and 25% of the prospective participants from strata of size ≥100 and <100 were systematically selected. A total of 1,247 workers were hence invited to join the study, of whom 807 workers (response rate = 64.7%) provided written consent and completed the questionnaire (n = 514, 143 and 150 for the three factories).

With written informed consent, participants self-administered the questionnaire in their factory’s hall, where they were assisted by fieldworkers. Participants were ensured that participation is absolutely voluntary and confidential, that no information would be released to the management. Participants were given ¥20 (about US$3.2) as a token of appreciation. The study was approved by the Ethics Committee of the Chinese University of Hong Kong.

### Measures

#### Background variables

Participants’ socio-demographic information (age, education level, gender, marital status and work-type) and length of working hours (per week) was collected.

#### Physical activity level

Participants were asked about the amount of time they spent on moderate or vigorous physical activity in the last seven days (“During the last 7 days, how much time did you spend on moderate and vigorous physical activities?”). Moderate exercise was described to the participants as “activities that require moderate physical effort and cause small increases in breathing or heart rate”. This question has been used in other published studies [62–64]. WHO (2010) [65] recommends people to perform 150 minutes of moderate or vigorous physical activities per week. In this study, we further classified those having less than 30 minutes of moderate or vigorous physical per week as having VLLPA.

#### Perceived job stress due to workload

The level of perceived job stress due to workload was assessed by using the 5-item Workload Subscale of the Job Stress Questionnaire [66], which has been used in some Chinese populations [67]. The items were rated on a seven-point Likert scale from “never” (1) to “always” (7). A sample item was “how often does your job leave you with little or no time to get things done?” Higher scores mean stronger perceived job stress. In this study, the Cronbach’s alpha of this subscale was 0.79.

#### Perceived social support

Two items were constructed for this study to assess levels of emotional and instrumental social support: “How often do you receive emotional support from others when you need it?” and “How often do you receive tangible help when you need it (e.g. in a situation of financial hardship)?” A four-point Likert scale, from “never” (1) to “always” (4), was used. The scores of the two items were added up to form the Perceived Social Support Scale (alpha in this study = 0.70). A higher score means better social support.

### Data Analysis

We created a binary variable for VLLPA: < = 30 minutes (69.6% of all participants) and >30 minutes (30.4% of all participants). Using this as the dependent variable, univariate odds ratios (ORu) were derived for all independent variables of this study. A multiple forward stepwise logistic regression model was fit (forward LR selection, entry criteria *p* = 0.10, removal criteria *p* = 0.20), using all significant background variables as candidates. Further analysis was conducted to test the significance of the associations between three independent variables (working hour, perceived job stress due to workload and perceived social support) and the dependent variable (VLLPA), adjusted for the significant background variables obtained from the stepwise modeling. Respectively 95% confidence intervals (CI) of the odds ratios were presented. SPSS 16.0 for Windows was used for data analysis; *p* values of <0.05 were taken as statistically significant.

## Results

### Background Characteristics

The results are summarized in Table 1. The mean age of the participants was 31.2 years (SD = 7.95 years; median = 31.0 years; range = 17 to 59 years). Over 60% of them were female (62.6%), having attained only junior high school or below (68.4%) and currently married (69.1%). The majority of them (94.1%) were production line workers, whilst the rest were office workers. The mean working hours per week was 57.56 (SD = 13.78), with 10.8% of them working for >76 hours per week.

**Table 1:** Descriptive statistics by gender.

	Total	Male	Female	*p* value^a^
**Background factors**				
**Gender**	807(100)	302(37.4)	505(62.6)	
**Age group (years)**				0.002
< 20	42(5.2)	15(5.0)	27(5.3)	
20–29	307(38)	137(45.4)	170(33.7)	
30–39	325(40.3)	98(32.5)	227(45.0)	
≥ 40	133(16.5)	52(17.1)	81(16.0)	
**Highest education level attained**				<0.001
Primary	53(6.6)	9(3.0)	44(8.7)	
Junior high school	499(61.8)	155(51.3)	344(68.1)	
Senior high school	218(27.0)	119(39.4)	99(19.6)	
Bachelor degree or higher	37(4.6)	19(6.3)	18(3.6)	
**Current marital status**				0.001
Single	233(28.9)	110(36.5)	123(24.4)	
Divorced/ widowed	16(2.0)	7(2.3)	9(1.8)	
Married	558(69.1)	185(61.2)	373(73.8)	
**Type of worker**				0.063
Production line	759(94.1)	277(91.7)	482(95.4)	
Office	22(2.7)	13(4.3)	22(2.7)	
Others	26(3.2)	12(4.0)	26(3.2)	
**Working hours (per week)**				0.020
≤48	244(30.2)	75(24.8)	169(33.5)	
49–60	302(37.4)	113(37.4)	189(37.4)	
61–69	68(8.4)	35(11.6)	33(6.5)	
70–75	106(13.1)	45(14.9)	61(12.1)	
≥76	87(10.8)	34(11.3)	53(10.5)	
**Psychosocial factors**				
**Perceived Social Support Scale (Mean, SD)**	5.7(1.37)	5.55(1.43)	5.79(1.33)	0.015
**Workload Subscale score (Job stress) (Mean, SD)**	17.57(7.99)	19.15(8.20)	16.63(7.72)	<0.001
**Physical activity level (moderate/vigorous)**				0.060
None	289(35.8)	99(32.8)	190 (37.6)	
≤0.5 hr/week	273(33.8)	91(30.1)	182(36.0)	
0.5–1 hr/week	135(16.7)	59(19.5)	76(15.0)	
1–1.5 hr/week	27(3.3)	13(4.3)	14(2.8)	
1.5–2 hr/week	20(2.5)	11(3.6)	9(1.8)	
2–2.5 hr/week	27(3.3)	14(4.6)	13(2.6)	
>2.5 hr/week	36(4.5)	15(5.0)	21(4.2)	

^a^ Statistical test: *t* test for continuous variable and χ2 test for categorical variables were used to analyze the gender differences.

### Physical Activity, Perceived Job Stress due to Workload and Perceived Social Support

It is alarming to see that over one third (35.8%) of the participants reported no moderate or vigorous activity per week at all (32.8% for males and 37.6% for females; Table 1). Furthermore, 69.6% had had only≤30 minutes moderate or vigorous physical activity per week (62.9% for males and 73.6% for females; *p* = 0.001). As mentioned, we defined this condition as VLLPA. The majority of the participants (95.4%) were unable to meet the level of moderate or vigorous physical activity (150 minutes per week), according to the Global Recommendation on Physical Activity of WHO (2010) and there was no significant gender difference (95.0% for males and 95.8% for females; *p* = 0.590). Weighted for stratification, the prevalence of VLLPA was 68.2%, which was very similar to the crude prevalence of 69.6%.

Compared to female participants, male participants were more likely to perceive higher level of job stress due to workload (*t* = 4.393, *df* = 805, *p*<0.001; Table 1), and lower social support (*t* = -2.44, *df* = 805; *p* = 0.015; Table 1).

### Factors Associated with VLLPA and Interaction Effects

Considering background factors, the results of the univariate analysis showed that females were more likely than males to have VLLPA (OR = 1.65; 95% CI: 1.21,2.24), while higher education attainment (junior high school: OR = 0.33, 95% CI: 0.14,0.80; senior high school: OR = 0.22, 95% CI: 0.09, 0.54; bachelor degree or higher: OR = 0.10, 95% CI: 0.03, 0.28; primary school as the reference group) and single marital status (OR = 0.63; 95% CI: 0.46, 0.88) were negatively associated with VLLPA. All these three variables (gender, education level and marital status) remained significant in the multiple stepwise logistic regression model (Table 2). Work type and working hour were however, statistically non-significant.

**Table 2:** Associations between background factors and “a very low level of physical activity” (i.e. having ≤30 minutes of moderate or vigorous physical activity in the last week).

	Row %	ORu (95% CI)	*p* value	ORm (95% CI)	*P* value
**Gender**					
Male	62.9	1.00		1.00	
Female	73.7	1.65 (1.21–2.24)	0.001	1.42 (1.03–1.95)	0.034
**Age group (years)**					
< 20	73.8	1.00		—	
20–29	61.9	0.58 (0.28–1.19)	0.136		
30–39	74.2	1.02 (0.49–2.11)	0.962		
≥ 40	75.2	1.08 (0.49–2.38)	0.858		
**Highest education level attained**					
Primary	88.7	1.00			
Junior high	72.3	0.33 (0.14–0.80)	0.014	0.38 (0.16–0.90)	0.029
Senior high	63.3	0.22 (0.09–0.54)	0.001	0.28 (0.11–0.68)	0.005
University or above	43.2	0.10 (0.03–0.28)	<0.001	0.13 (0.04–0.37)	<0.001
**Current marital status**					
Married	72.2	1.00		1.00	
Single	62.2	0.63 (0.46–0.88)	0.006	0.75(0.54–1.05)	0.090
Divorced/ widowed	87.5	2.69 (0.61–11.98)	0.194	2.65(0.59–11.95)	0.205
**Type of worker**					
Production line	69.4	1.00		—	
Office	72.7	1.17(0.45–3.04)	0.741		
Others	73.1	1.20(0.50–2.88)	0.692		

ORu: univariate odds ratios; ORm: multivariate odds ratios obtained from forward stepwise multivariate logistic regression using variables found to be significant in univariate analysis as candidate variables; 95%CI: 95% confidence interval.—: Variables with *p*>0.1 in the univariate analyses and were not used in the subsequent stepwise model.

In the univariate analysis, both perceived social support (OR = 0.87; 95% CI: 0.78, 0.97) and perceived job stress due to workload (OR = 0.98, 95% CI: 0.96, 0.99) were negatively associated with VLLPA (Table 3). Adjusted for the significant background variables, perceived social support, but not perceived job stress due to workload, remained statistically significant (OR = 0.88; 95% CI: 0.78, 0.98; Table 3).

**Table 3:** Work hour, perceived job stress due to workload and perceived social support as associated factors of “a very low level of physical activity” (i.e. having ≤30 minutes of moderate or rigorous physical activity in the last week).

	Row %	ORu (95% CI)	*p* value	AOR (95% CI)	*p* value
**Working hours per week**					
≤48	70.1	1.00		1.00	
49–60	70.5	1.02(0.71–1.48)	0.909	1.00(0.68–1.47)	0.998
61–69	72.1	1.10(0.61–2.00)	0.752	1.38(0.74–2.58)	0.315
70–75	65.1	0.80(0.49–1.29)	0.356	0.89(0.53–1.47)	0.639
≥76	69.0	0.95(0.56–1.61)	0.846	0.98(0.56–1.71)	0.941
**Workload Subscale score (Job stress)**	NA	**0.98(0.96–0.99)**	**0.011**	0.99(0.97–1.01)	0.184
**Perceived Social Support Scale score**	NA	**0.87(0.78–0.97)**	**0.016**	**0.88(0.78–0.98)**	**0.028**

ORu: univariate odds ratios; AOR: Adjusted odds ratios, odds ratios adjusting for all multivariately significant background variables listed in Table 2, including gender, highest education level and current marital status; 95%CI: 95% confidence interval; NA: Not applicable

**Fig 1:**
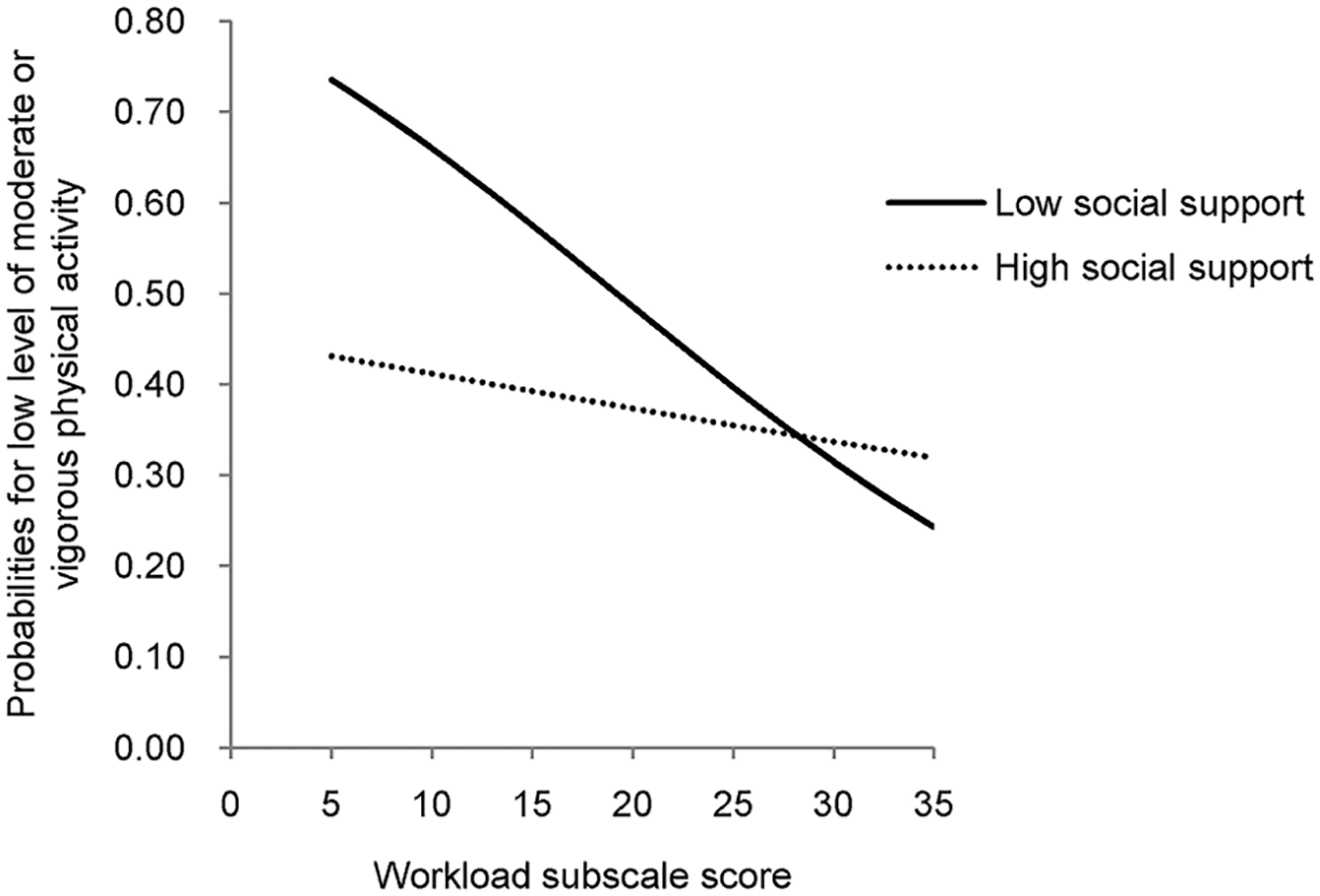
Interaction effects between perceived social support and job stress due to workload on probability of having a very low level of physical activity. Solid line represents high social support, dotted line represents low social support. Two lines crossing over each other indicates that job stress due to workload interacted significantly with perceived social support (*p* = 0.044). Very low level of physical activity indicates having had ≤30 minutes of moderate/vigorous physical activity in the last week.

Adjusted for significant background variables, perceived job stress due to workload interacted significantly with perceived social support (*p* = 0.044; Table 4) to determine VLLPA. The results implied that stronger perceived job stress due to workload was negatively associated with VLLPA. The strength of association (facilitation effect) was however, stronger among those with weaker perceived social support than among those with stronger social support (Fig 1). Perceived support hence moderated the relationship between perceived job stress due to workload and VLLPA.

**Table 4:** Interaction between perceived social support and perceived job stress due to workload onto having “a very low level of physical activity” (i.e. having ≤30 minutes of moderate or vigorous physical activity in the last week).

	Beta	OR	95%CI	*p* value
(1) Workload Subscale score (Job stress)	-0.10	0.91	0.83–0.98	0.021
(2) Social Support Scale score	-0.39	0.68	0.51–0.90	0.008
(1) x (2)	0.01	1.01	1.00–1.03	0.044

Adjusted for all significant background variables listed in Table 2.

## Discussion

In this study, it is very alarming to find that the majority of the participants, who were migrant factory workers, were not physically active. Only 5.4% of them met the WHO recommendation, whilst 35% reported no moderate or vigorous physical activity in the last week at all. Previous studies have reported that 66.3% to 70.6% of the general population in China met the WHO recommendation [68,69]. Therefore, the level of physical activity among the sampled factory workers was very much lower than that of the general population in China. As most of the participants came from rural areas where the prevalence of physical inactivity (below WHO recommendation) was as low as 21.9% [69], a dramatic change in life style should have occurred. It is known that urbanization leads to sedentary lifestyle [16]. We contended that such an effect on physical activity may have further been compounded by the sedentary job nature and some factory settings.

Corroborating with the results obtained from other studies (e.g. Muntner, 2005) [69], female workers were more likely than male workers to have even less moderate or vigorous physical activity. Like other studies (e.g. Parks, 2003)[70], we also found that lower education levels were associated with VLLPA, possibly due to association between education level and health value/literacy [71,72]. In literature as well as in this study, married individuals tend to be less physically active, as compared to their single counterparts [73–75]. Therefore, health promotion should target female, married, and less educated migrant factory workers. Considerations should take into account their interests when planning health promotion for physical activities.

The job nature of factory workers is a potential structural barrier that may lead to physical inactivity. About two-third of the sampled factory workers worked over 50 hours per week and their job task was extremely sedentary and repetitive. Within such long working hours, there is almost no moderate or vigorous physical activity. Interpersonal factors are also important. Results of this study’s adjusted analysis and those obtained by other studies [29,43,44,45,46] supported the hypothesis that weaker perceived social support would be associated with less physical activity. However, factory workers in China tend to have severely weakened social relationship [76,77]. It may be difficult for participants to find friends to exercise together, as their friends are also likely to be factory workers, and they may find it too busy to meet with each other. The finding gives an insight that physical activity may be promoted via strengthening perceived social support, such as organizing group physical activities.

We found that stronger perceived job stress was negatively associated with VLLPA. The finding supports the ‘adaptive coping’ hypothesis that workers would perform some exercises to alleviate stress, instead of our initial hypothesis that perceived job stress due to workload would prevent workers from exercising. In literature, the findings of the association between job stress and physical activity have been mixed [25,26,27,29], as physical exercise could be used as a positive coping response of perceived stress or sedentary lifestyle could become a negative coping response of perceived stress [25,78,79]. It is hence possible to promote physical activities as an effective means of stress reduction when planning health promotion programs.

Unexpectedly, we found that long working hour was not associated with VLLPA. No study has investigated such an association in this population. Although there were some variations, the majority of the workers were working long hours (64.2% worked >50 hours per week), so that most of them may have little time and energy to do exercise, taking aside time required for daily mandates. The finding is consistent with the interpretation that job stress due to workload was not a risk factor but a protective factor against having “a very low level of physical activity”. The important message is that long working hour and perceived job stress due to workload would not prevent workers from performing physical activity, and should not be used as reasons for not promoting physical activity among them.

The observed significant interaction between perceived job stress and perceived social support onto VLLPA suggests that positive association between perceived stress and physical activity level was more obvious among those with weaker perceived social support than among those with stronger perceived social support. It is possible that factory workers used physical activity as a means of stress reduction, while this adaptive coping mechanism was more common and important among those with lower perceived social support. It implies that promoting physical activity as a means of stress reduction would be more effective among those with lower levels of social support. It is possible that those with lower social support have a stronger need to find ways to relieve their stress, while performance of exercise serves as one of those means.

The study has some limitations. The data were obtained from only three factories so that generalization to the many other factories in Shenzhen and other cities needs to be made with caution. Furthermore, these three factories manufactured electronic products and only belonged to the light industry. Extra caution is hence required when making generalization. The study population of migrant factory workers of the light industry is however, still very sizable, as such factories make up about half of all factories in Shenzhen and a large proportion of those in other Chinese coastal cities. Analysis on inadequate physical activity was not performed as the majority (over 90%) of the sample showed physical inactivity. Instead, factors of VLLPA were identified. We may have omitted other important potential factors and confounders such as cognitions for physical activity. The study was therefore as an exploratory one to look at the size of the problem and some potential factors related to work. Physical activity was self-reported by using a single question (that has also been used in other studies), which have not been validated for this study. The scale items of perceived social support were self-constructed and have not been validated. Self-reporting responses may also be subjected to reporting bias due to social desirability but the direction of the bias would increase estimated prevalence of physical inactivity further.

## Conclusion and Implications

In conclusion, it is likely that internal migrant factory workers of the light industry in mainland China, especially those who were females, lowly educated, being single and perceived low social support, have adopted an extremely sedentary lifestyle. They may need to live such a highly sedentary life in factory setting for many years in the future, inflating their risks of NCD and contribute heavily to disease burden in China. Without effective interventions, related negative public health consequences would soon emerge, as there are strong and consistent evidences showing that physical inactivity is strongly predictive of ischemia heart disease, ischemic stroke, diabetes, colon cancer, obesity, cognitive impairment, dementia, and depression [5,9,80,81].

Health promotion for this population is greatly warranted but may have been overlooked by health workers and policy makers in China. According to our observations and literature review, there is no consistent health promotion program targeting this special population in China. Interpersonal and structural factors need to be taken into account when promoting physical activities. Increase in social support is potentially important. Creation of supportive social networks for participation in sport activities may be considered. Further needs assessments and pilot interventions are greatly warranted to design such programs.

Policies should be made to enhance factory managements’ recognition of importance of factory workers’ health and risks involved, as well as their responsibility and role in promoting factory workers’ health. Health promotion through social marketing should be provided to migrant factory workers by factories’ management, one of the most important stakeholders. Managers need to be made aware of the unfavorable sedentary life-style of their factory workers and negative implications of physical inactivity onto their health and subsequent productivity. Such intervention programs may include organization of regular events for promoting individual and group exercises and sport activities, provision of better exercise facilities, creation of routine time-slots during working hours for physical activity (e.g. 15 minutes of group exercise daily before work starts), and provision of incentives for exercising (e.g. gifts and small monetary incentive).

The very low level of physical activity observed in this study may be better understood by inspecting related context and setting. According to the socio-ecological model, factors of individual, inter-personal and structural levels are all important determinants of health-related behaviors [82–84]. The model applies well to explain variations in physical activity level [85,86], which is significantly associated with distance from free sport facilities in the community [87]. Factories in Shenzhen are mostly built in isolated and remote areas to save cost. There is in general a lack of sport facilities inside the factory and in close-by accessible areas. There are hence structural reasons that may partially explain the observed low physical activity level among factory workers in China. This contention, if proven true, has important policy implications on improving physical activity facilities requirements of factories in China.

Last but not the least, the Ministry of Health in China need to review relevant polices to protect health of factory workers; factory owners should fulfill their social responsibility in improving factory workers’ health. Health promotion of physical activity among migrant workers of the light industry in China at cognitive, inter-personal and structural levels is greatly warranted.
